# A matter of freedom? Right-wing populist frames of Austria’s smoke-free policy

**DOI:** 10.1093/eurpub/ckac131.540

**Published:** 2022-10-25

**Authors:** M Andreas

**Affiliations:** Heidelberg University, Center for Preventive Medicine and Digital Health, Mannheim, Germany

## Abstract

**Background:**

Even though right-wing populist parties (RWPP) are increasingly influential in Europe, their position on public health has hardly been researched. I aimed to fill this research gap by focusing on the case of the FPÖ (Freiheitliche Partei Österreichs) and its opposition to Austriás smoke-free policy. Understanding RWWP positions on health policy can help to prevent detrimental influences on health policy making. Therefore, this project aimed to investigate how the FPÖ framed Austria’s smoke-free policy and whether newspaper articles reflect these frames.

**Methods:**

Online archives of the three most-read Austrian newspapers (Standard, Kurier, Kronenzeitung) were searched for articles on the policy published in November 2019, when the policy was implemented. Furthermore, speeches by FPÖ politicians in the parliamentary debate on the policy in June 2019 were identified via the parliamentary archive. Drawing on 4 speeches by FPÖ politicians and 29 newspaper articles, I used frame analysis to answer the research question.

**Results:**

The analysis yielded that FPÖ politicians used authoritarian, populist, and libertarian frames to argue against the implementation of the smoke-free policy. Thus, the policy was portrayed as not being in the interest of the people, restricting personal and economic freedom, and as elitist. These frames were not reflected in newspaper reports that mainly focused on the practical aspects of the policy implementation. However, in contrast to politicians supporting the policy, FPÖ politicians were overrepresented in newspaper reports representing 47% of political actors cited in newspaper articles.

**Conclusions:**

Authoritarian, populist, and libertarian frames were used by the FPÖ in opposition to the smoke-free policy. While these frames were not replicated by Austrian newspapers, RWP politicians were overrepresented in articles on the policy. These findings illustrate the importance of effective counter-framing by health advocates.

**Key messages:**

• RWPP used authoritarian, populist, and libertarian frames to oppose Austria’s smoke-free policy.

• Even though RWPP frames were not replicated in newspaper articles, RWPP politicians were overrepresented in reports on the policy.

